# Novel CARMIL2 Mutations in Patients with Variable Clinical Dermatitis, Infections, and Combined Immunodeficiency

**DOI:** 10.3389/fimmu.2018.00203

**Published:** 2018-02-09

**Authors:** Anas M. Alazami, Maryam Al-Helale, Safa Alhissi, Bandar Al-Saud, Huda Alajlan, Dorota Monies, Zeeshan Shah, Mohamed Abouelhoda, Rand Arnaout, Hasan Al-Dhekri, Nouf S. Al-Numair, Hazem Ghebeh, Farrukh Sheikh, Hamoud Al-Mousa

**Affiliations:** ^1^Department of Genetics, King Faisal Specialist Hospital and Research Centre, Riyadh, Saudi Arabia; ^2^Saudi Human Genome Program, King Abdulaziz City for Science and Technology, Riyadh, Saudi Arabia; ^3^Department of Pediatrics, Prince Sultan Military Medical City, Riyadh, Saudi Arabia; ^4^Department of Pediatrics, Allergy and Immunology Section, King Faisal Specialist Hospital and Research Centre, Riyadh, Saudi Arabia; ^5^Department of Medicine, Allergy and Immunology Section, King Faisal Specialist Hospital and Research Centre, Riyadh, Saudi Arabia; ^6^Alfaisal University, Riyadh, Saudi Arabia; ^7^Stem Cell & Tissue Re-Engineering Program, King Faisal Specialist Hospital and Research Centre, Riyadh, Saudi Arabia

**Keywords:** CARMIL2, immunodeficiency, whole exome sequencing, autosomal recessive, RLTPR, dermatitis, infection, CD28

## Abstract

Combined immunodeficiencies are a heterogeneous collection of primary immune disorders that exhibit defects in T cell development or function, along with impaired B cell activity even in light of normal B cell maturation. CARMIL2 (RLTPR) is a protein involved in cytoskeletal organization and cell migration, which also plays a role in CD28 co-signaling of T cells. Mutations in this protein have recently been reported to cause a novel primary immunodeficiency disorder with variable phenotypic presentations. Here, we describe seven patients from three unrelated, consanguineous multiplex families that presented with dermatitis, esophagitis, and recurrent skin and chest infections with evidence of combined immunodeficiency. Through the use of whole exome sequencing and autozygome-guided analysis, we uncovered two mutations not previously reported (p.R50T and p.L846S*fs*) in CARMIL2. Real-time PCR analysis revealed that the biallelic frameshift mutation is under negative selection, likely due to nonsense-mediated RNA decay and leading to loss of detectable protein upon immunoblotting. Protein loss was also observed for the missense mutation, and 3D modeling suggested a disturbance in structural stability due to an increase in the electrostatic energy for the affected amino acid and surrounding residues. Immunophenotyping revealed that patient T_reg_ counts were significantly depressed, and that CD4^+^ T cells were heavily skewed towards the naïve status. CD3/CD28 signaling impairment was evidenced by reduced proliferative response to stimulation. This work broadens the allelic heterogeneity associated with CARMIL2 and highlights a deleterious missense alteration located outside the leucine-rich repeat of the protein, where all other missense mutations have been reported to date.

## Introduction

The term “combined immunodeficiencies” (CID) refers to a heterogeneous collection of immunodeficiencies that exhibit defects in T cell development or function. Due to dysfunction of CD4^+^ helper T cells, B cell activity is also affected even if B cell maturation proceeds as normal (1). Hence both arms of the lymphocyte response system (cellular and humoral) are affected, giving the disorder its “combined” designation. CIDs are the result of inherit flaws in the lymphocyte differentiation pathway. SCID, a specialized form of CID that is defined by the absence of detectable autologous (host origin) T cells in blood and lymphoid tissues, can arise mechanistically due to apoptosis of hematopoietic progenitor cells, defective cytokine or T-cell receptor (TCR) signaling, or other pathways (2). Such patients present very early in life with interstitial pneumonia, failure to thrive, candidiasis, and chronic diarrhea, and a total of 16 genes thus far are implicated in SCID pathogenesis (3).

Over the past 25 years, there has been a jump in the identification and characterization of a growing number of patients who, despite presenting with the repeated opportunistic infections that are generally observed with SCID, nevertheless exhibit a significant number of autologous circulating T cells (1, 4). Such individuals are classed as CID, defined clinically as patients with partial T and B cell abnormalities who survive after the age of 2 years without immunologic reconstitution. They frequently present with a prominent skin rash, enlarged lymph nodes, and a normally sized thymus. Over 40 different molecular defects can result in CID (3), and the list continues to expand due to the availability and power of next-generation sequencing along with advances made in functional assays.

CARMIL2 (RLTPR) mutations have been recently reported by three independent groups to cause a novel autosomal recessive, primary immunodeficiency disorder with variable phenotypic presentations. Wang et al. described six patients from three unrelated families with biallelic loss-of-function mutations in CARMIL2 affecting the CD28-responsive pathway in T cells and the BCR-responsive pathway in B cells. These patients have cutaneous and pulmonary allergy, as well as various bacterial and fungal infectious diseases including invasive tuberculosis and mucocutaneous candidiasis (5). Sorte et al. described another four patients from three Norwegian families presenting with a common skin phenotype of warts, molluscum contagiosum, and dermatitis since early childhood, along with various other immunological features. All patients were homozygous for a CARMIL2 missense founder mutation (6). Schober et al. reported four human patients with Epstein–Barr virus (EBV)-related disseminated smooth muscle tumors that carried two homozygous CARMIL2 mutations associated with defective CD28-mediated TCR co-signaling and impaired cytoskeletal dynamics (7).

Due to the autosomal recessive nature of most CID- (and SCID-) associated genes, there is a compelling likelihood that mutations in such genes will be more prevalent in countries where consanguinity is more widespread (8, 9). Here, we utilized whole exome sequencing (WES) and other assays to genetically and functionally characterize three consanguineous multiplex Saudi families with CARMIL2 mutations, presenting with dermatitis, recurrent skin abscesses and chest infections.

## Materials and Methods

### Patients

**Figure 1:**
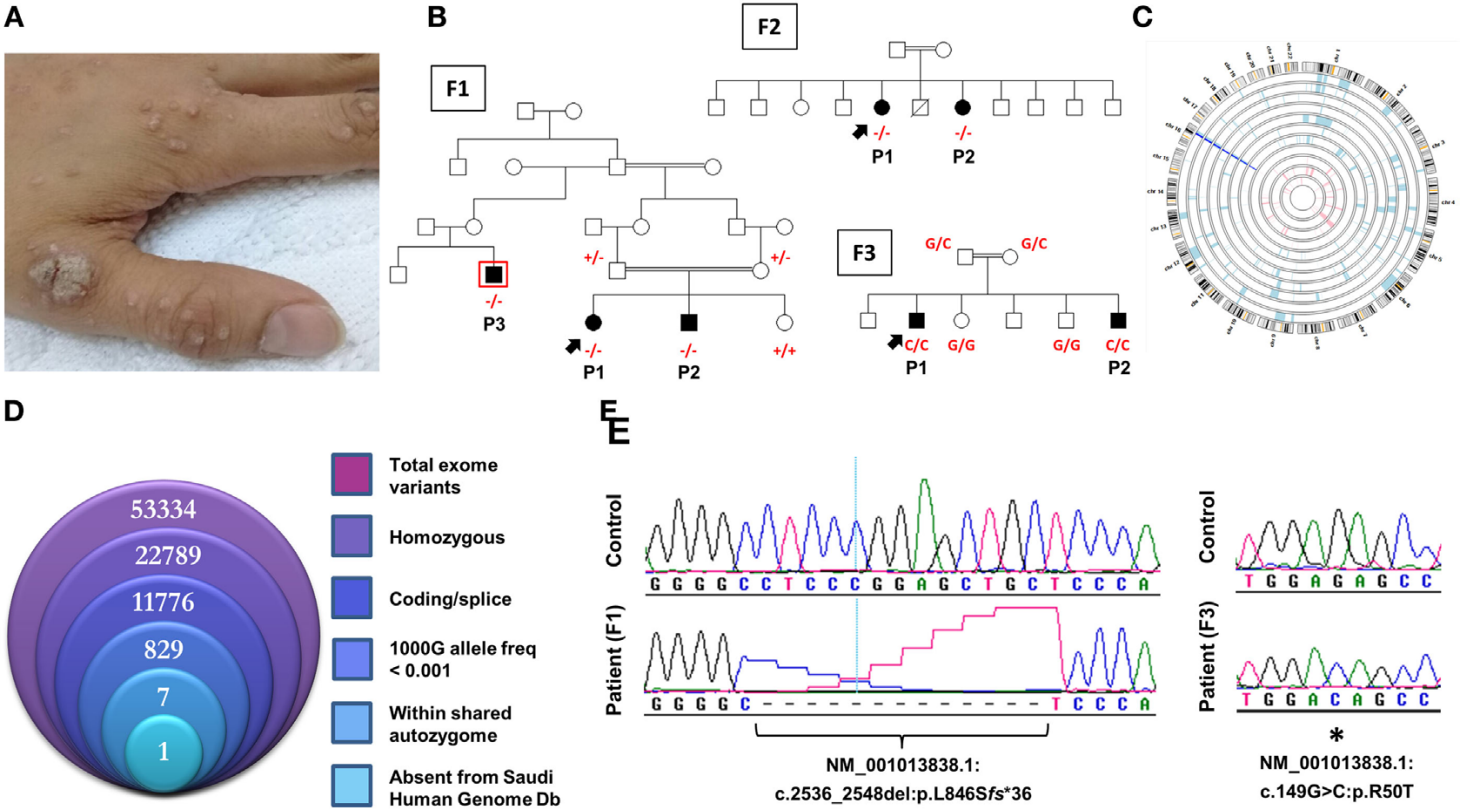
Novel *CARMIL2* mutations in patients with combined immunodeficiency. **(A)** Index case from F3 with disseminated warts infection. **(B)** Pedigrees of the three consanguineous families in this study. Index cases are indicated by arrows, and genotypes (in red) are provided for all family members we had access to. Patients are identified with the abbreviations used in the text. Exome sequencing was performed on patient F1P3, who is boxed in red. **(C)** Circular representation of chromosomes indicating regions of homozygosity (ROH) in affected (pale blue) and unaffected (pink) individuals. Dark blue denotes the only region that contains ROH across all patients, which coincides with the *CARMIL2* locus. **(D)** Stacked Venn diagram illustrating the total number of variants observed with exome capture, following inclusion of the indicated filters. **(E)** Sequence chromatograms of the frameshift and nonsense *CARMIL2* mutations, with control sequences for reference.

A total of seven patients from three unrelated families with ill-defined suspected primary immunodeficiency diseases were recruited to the study from immunodeficiency clinics at King Faisal Specialist Hospital & Research Centre (KFSHRC) and Prince Sultan Military Medical City (Figure 1B). All research involving patient materials was approved by the institutional review board at KFSHRC, RAC #2080 025. Written consent was obtained from patients or their guardians and supported by the NSTIP strategic technologies program of Saudi Arabia (KACST 14-MED316-20).

### Sanger Sequencing

Genomic DNA was extracted from whole blood samples. Primers were designed from within the intronic regions to span the exon/intron boundaries, plus the full exonic sequence, with 5′ tagged M13 sequences. DNA was sequenced with the BigDye Terminator. To rule out the possibility that the mutations were unreported normal variants segregating in the local population, all mutations were interrogated against the Saudi Human Genome Database in addition to a separate database of 816 ethnically matched exomes.

### Genomewide High-Density Genotyping

Genomewide SNP genotyping and homozygosity mapping was performed using the AxiomGWH SNP Chip platform (Affymetrix). The AxiomGWH genotyping files (containing 587,352 SNP calls each) were fed into the AutoSNPa software to determine the autozygome. A size limit of 2.0 Mb for calling autozygous intervals was implemented. This allowed us to take advantage of autozygosity without succumbing to short identical-by-state regions of homozygosity which are abundant in populations with high consanguinity levels. Both raw (cel) and processed (txt) genotyping files are deposited in the ArrayExpress database at EMBL-EBI,^1^ accession number E-MTAB-6440.

### Whole Exome Sequencing

100 ng of DNA from the index case was treated to obtain the Ion Proton AmpliSeq library. Briefly, DNA was amplified in 12 separate wells using Exome Primer Pools, AmpliSeqHiFi mix (ThermoFisher, Carlsbad, CA, USA), and 10 amplification cycles. All 12 PCR pools were combined in one well and subjected to primer digestion, incubated with FuPa reagent (ThermoFisher). Amplified exome targets were ligated with Ion P1 and Ion Xpress Barcode adapters. After purification, libraries were quantified using quantitative PCR with the Ion Library Quantification Kit (ThermoFisher). The prepared exome library was further used for emulsion PCR on an Ion OneTouch System and templated Ion Sphere particles were enriched using Ion OneTouch ES, both procedures following the manufacturer’s instructions. The template-positive Ion PI Ion Sphere particles were processed for sequencing on the Ion Proton instrument (ThermoFisher). The total number of bases was 16.5 Gbp (89.24 million reads with median read length of 184 bp); 97.8% of the bases (from 98.9% of the reads) were aligned to the reference genome, achieving 99.7% coverage of the target exonic regions at an average depth of 258. Reads were mapped to UCSC hg19,^2^ and variants identified using the Saudi Human Genome Program pipeline (10). The final step of the analysis was filtering of the resultant data, where only novel homozygous coding or splicing variants within candidate autozygous intervals were considered. The exome filtering process is summarized in Figure 1D. The BAM file for WES is deposited in the European Nucleotide Archive,^3^ study accession number PRJEB24299.

### RNA Expression Analysis

Lymphoblastoid cell lines were established from indicated patients using standard protocols. RNA extraction was followed by cDNA synthesis and real-time reverse-transcriptase PCR (Applied Biosystems 7500 Fast Real-Time PCR System) using exon–exon spanning primers to avoid genomic DNA amplification. Primer sequences were 5′-TGCAGTCCATCAGATCCAAG-3′ and 5′-GCACCGACTGACACAATTCA-3′. QuantiTect SYBR Green mix (Qiagen, Limburg, Netherlands) was used for quantification, and the deltaCt method was utilized to calculate fold difference compared with normal controls (with GAPDH for internal normalization). All reactions were performed using at least two independent runs, in triplicate each time, then normalized to GAPDH using the built-in software (v2.0.5) to generate the relative quantification.

### Immunoblotting

Lysates from lymphoblastoid cell lines were prepared using RIPA buffer (Sigma, St. Louis, MO, USA) containing a protease inhibitor cocktail mix (Roche, Indianapolis, IN, USA). Samples were normalized by measuring on a Smart-Spec Plus spectrophotometer (Bio-Rad, Hercules, CA, USA) using the Protein Assay Dye Reagent (Bio-Rad), electrophoresed on an SDS-PAGE (National Diagnostics, Atlanta, GA, USA), and transferred onto a PVDF membrane (GE Healthcare, Waukesha, WI, USA). The HRP-conjugated secondary antibodies were detected by SuperSignal WestPico Chemiluminescent (Thermo Scientific, Rockford, IL, USA). The following primary antibodies were used: rabbit anti-RLTPR/CARMIL2 (ab122717) and mouse anti-beta Actin (ab8226) (Abcam, Cambridge, MA, USA).

### 3D Modeling

**Figure 2:**
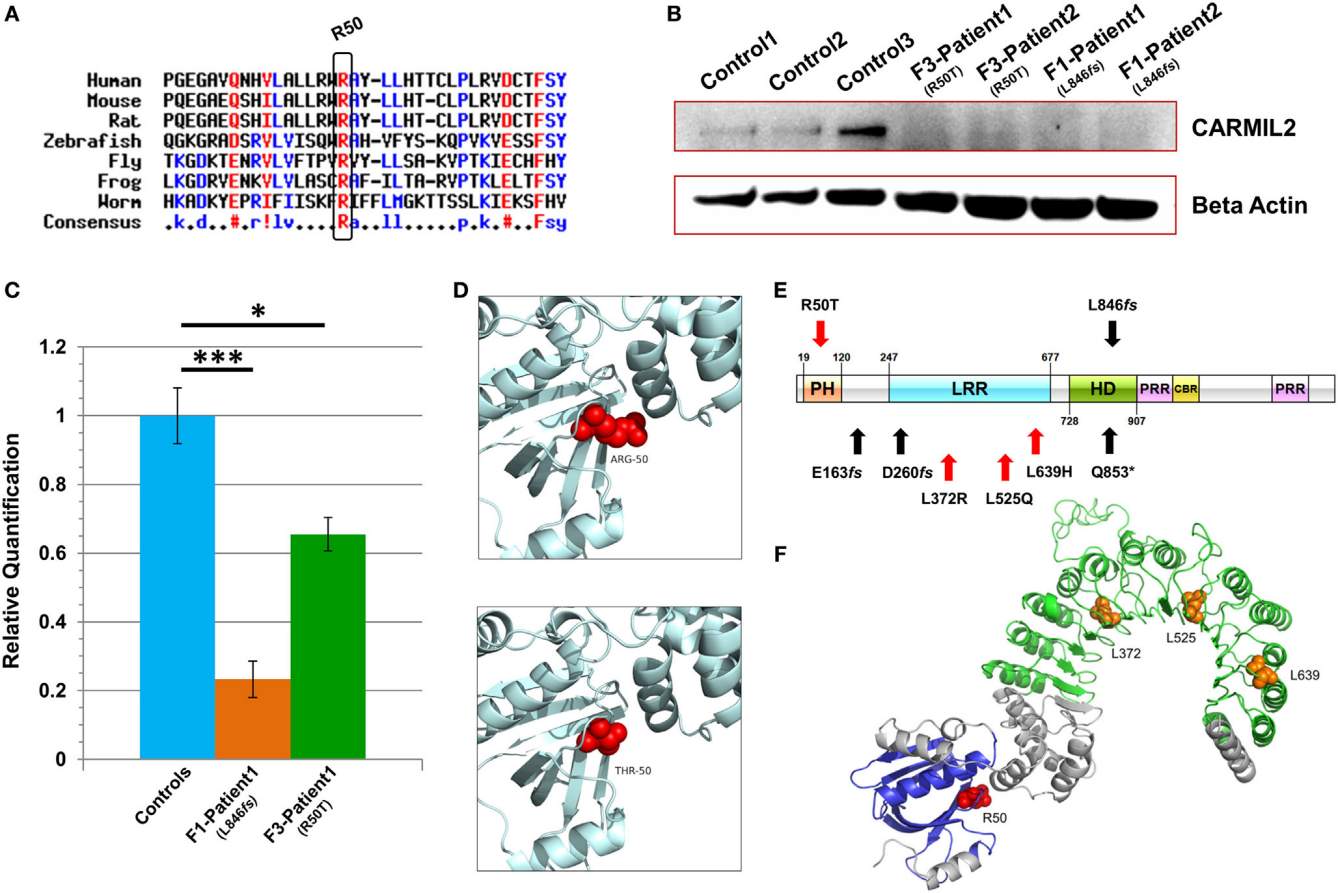
Characterization of CARMIL2 mutations. **(A)** Protein sequence alignment reveals that the Arg-50 residue is highly conserved across species. **(B)** Immunoblot reveals absence of detectable CARMIL2 protein in lymphoblastoid cells from patients with the missense and frameshift mutations. For the latter, no bands were found at the calculated molecular weight of the truncated protein (Figure S3 in Supplementary Material). The beta actin lane serves as a loading control. **(C)** Real-time RT-PCR data for CARMIL2 expression levels in lymphoblastoid cells from the index cases for families F1 and F3 versus combined data from two normal controls. Asterisks indicate significance levels (*p < 0.05, ***p < 0.001; unpaired Student’s *t*-test). Error bars indicate SEMs. Data were acquired through two independent experiments conducted in triplicate. **(D)** The 3D homology models established using the native and mutant amino acids at position 50. **(E)** Schematic representation of the CARMIL2 protein. Previously reported mutations are listed under the schematic, and those from this study are listed above. Missense mutations are indicated in red, and frameshift in black. The depicted domains include the pleckstrin homology domain (PH), the leucine-rich repeat (LRR), the homodimerization domain (HD), the proline-rich regions (PRR), and the capping protein binding region (CBR). Domain boundaries are approximate and are based on homology with known CARMIL1 domains (12) as well as previous CARMIL2 domain delineation (13). **(F)** 3D modeling of the N-terminal half of native CARMIL2, with the PH domain in blue and the LRR horseshoe region in green. All previously reported missense mutations (orange) have involved leucine residues within the LRR, whereas the one reported in this study (red) is an arginine that does not show interaction with that domain.

A homology model was built using the SWISS-MODEL (11) template library (SMTL version 2017-06-07, PDB release 2017-06-02), and the best structure template matching the target sequence was selected (4K17.2.A). The model starts with Thr-4 and shows that GLY189-ASN707 (which largely overlaps with the leucine-rich repeat, Figure 2E) has an overall planar horseshoe shape (Figure 2F). Arg-50 was altered to Thr-50 with the native side-chain being replaced by the mutant rotamer, which yielded the lowest score according to the lowest energy (fewest collisions and most favorable hydrogen bonds). Energy computations for the structures containing the native and mutant residues, respectively, were done with the GROMOS96 43B1 parameters set, without reaction field.

### Flow Cytometry

Frozen PBMCs were thawed, washed then left to settle for 2 h in complete medium (RPMI supplemented with 10% fetal calf serum, penicillin, and streptomycin). For cell surface antibodies, PBMCs were incubated for 30 min on ice with directly labeled antibodies, washed and resuspended in PBS and DAPI as a viability dye to exclude nonviable cells. Intracellular staining of FOXP3 was performed using a kit (eBioscience/Thermo Scientific) as per the manufacturer’s instructions. For the T cell proliferation assay, cells were labeled with CSFE (Invitrogen) for 15 min at 37°C, washed three times in complete medium, then incubated (25,000 cells/well) with CD3/CD28 beads (Invitrogen) for 72 h. Data were acquired using an LSR II Flow Cytometer using the BD FACSDiva operating software. Positive staining was considered based on the negativity of an isotype control and a minimum of 20,000 events were recorded for all samples. Antibodies used were AmCyan-anti-CD3, APC-anti-CD4, PerCP-Cy5.5-anti-CD4, Pacific Blue-anti-CD8, PE-Cy7-anti-CD8 (all BD Biosciences), FITC-anti-CD45RO, PE-anti-CD27 (DAKO), and APC-anti-CD25, PE-Cy7-anti-CD127 (eBioscience).

## Results

### Patients

F1P1 and F1P2 are two siblings born to consanguineous Saudi parents (Figure 1B). F1P1 is a 13-year-old girl with history of persistent dermatitis associated with recurrent skin abscesses since the age of 2 years secondary to *Staphylococcus aureus* infections. She developed progressive dysphagia. Upper gastrointestinal endoscopy showed chronic active esophagitis and esophageal candidiasis. She had no recurrent chest infections. Immunological workup showed poor specific antibody response to tetanus vaccination and variable poor T-cell response to mitogen and antigen stimulation (Table 1). F1P2 is a 9-year-old boy who started to complain of dermatitis and skin abscesses at the age of 5 years. He complained also of dysphagia secondary to chronic active esophagitis. F1P3 is a 29-year male (cousin of F1P1 and F1P2) who was following in our hospital for the last 20 years with history of persistent dermatitis, skin abscesses, and recurrent chest infections with secondary bronchiectasis since the age of 1 year. He complained of persistent dysphagia. Upper gastrointestinal endoscopy showed evidence of chronic active esophagitis with secondary Schatzki ring and hiatus hernia. EBV viremia was documented without any EBV-related clinical manifestation. Immunological work up showed poor antibody response to tetanus vaccine and poor T-cell response to mitogens and antigen stimulation (Table 1).

**Table 1:** Clinical and immunological features of CARMIL2 patients.

	F1P1	F1P2	F1P3	F2P1	F2P2	F3P1	F3P2	Normal range
Age (years)	13	9	29	34	28	12	4	
Sex	F	M	M	F	F	M	M	–
Onset of illness	3 years	5 years	1 year	2 years	9 years	3 months	2 weeks	–
Age at diagnosis	10	8	28	34	28	11	3	–
Eczema	Y	Y	Y	No	Y	Y	Y	–
Recurrent skin abscesses	Y	Y	Y	Y	Y	Y	Y	–
Chest infection	No	No	Y	Y	Y	Y	Y	–
Bronchiectasis	No	No	Y	Y	Y	Y	No	–
Chronic diarrhea	No	No	No	No	No	No	Y	–
Other clinical presentations	Dysphagia, esophageal stenosis, chronic active esophagitis, and candidiasis	Dysphagia and chronic active esophagitis	Dysphagia, chronic active esophagitis, Schatzki ring, and hiatus hernia	Dysphagia and *Candida* esophagitis	Pneumonectomy	Not done	Dysphagia and eosinophilic esophagitis Severe colitis and crypt abscesses	
EBV PCR (copies/ml)	ND	ND	57,260	35,460	ND	ND	ND	
Other infections	Skin *Staphylococcus aureus* *Candida albicans* esophagitis	Skin *S. aureus*	Skin *S. aureus*	*C. albicans* esophagitis	Dental	Skin *S. aureus* Warts Scalp fungal infection (*Aspergillus niger*) Chronic suppurative otitis Media anterior Nasal vestibulitis Cervical lymphadenitis	Chronic suppurative otitis media Warts Scalp fugal infection	
Other clinical presentations				Suspected hydatid cyst (negative biopsy)		Food allergy, asthma, and allergic rhinitis	Asthma and food allergy	
WBC (10^9^/L)	6.6	6.46	8.6	6.23	4.07	17.5	17.1	^a^
Neutrophils (10^9^/L)	3.2	1.95	6.02	4.48	2.2	6.3	5.9	^a^
Lymphocytes (10^9^/L)	2.6	3.82	1.98	1.13	1.38	6.5	7.2	^a^
Eosinophils (10^9^/L)	0.31	0.25	0.49	0	0	3.2	2.2	^a^
Monocytes (10^9^/L)	0.39	0.45	0.03	0.57	0.46	1.5	1.6	^a^
Hemoglobin (g/L)	115	114	121	100	130	140	114	^b^
Platelet	259	346	331	265	203	434	808	150–450 10^9^/L
CD3^+^	1,839	3,758	1,689	1,202	1,530	2,578	2,944	^c^
CD3^+^/CD4^+^	1,161	2,131	761	623	876	1,307	2,234	^c^
CD3^+^/CD8^+^	547	1,440	740	552	605	1,088	655	^c^
CD19^+^	367	843	423	46	10	947	1,506	^c^
CD16^+^/CD56^+^	333	194	21	31	21	87	527	^c^
CD3 CD45RA %	56	71	ND	48	39	ND	ND	–
CD4 CD45RA %	44	47	ND	31	23	ND	ND	–
CD3 CD45R0 %	12	7	ND	34	43	ND	ND	–
CD4 CD45RO %	8	5	ND	22	36	ND	ND	–
IgG (g/L)	15.3	10.4	14.9	6.8	10.4	11.1	5.83	^d^
IgA (g/L)	2.87	1.6	5.17	<0.50	2.77	3.25	2.21	^d^
IgM (g/L)	2.52	1.48	1.69	0.29	3.74	1.72	1.00	^d^
IgE (KU/L)	<2	44	1,153	<2	<2	>5,000	<2	^d^
Tetanus toxoid IgG (mg/L) (prevaccination)	2.17	ND	8	ND	0.59	0.48	0.85	
Tetanus toxoid IgG (mg/L) (postvaccination)	2.8	ND	10	ND	0.7	0.68	0.35	
Tetanus toxoid IgG1 (mg/L) (prevaccination)	<0.67	ND	6.5	<0.67	<0.67	ND	ND	
Tetanus toxoid IgG1 (mg/L) (postvaccination)	<0.67	ND	9.2	<0.67	<0.67	ND	ND	
Pneumococcal cap polysaccharide IgG (mg/L) (prevaccination)	90	ND	90	12.3	160	46.9	<3.3	–
Pneumococcal cap polysaccharide IgG (mg/L) (postvaccination)	>270	124	>270	8.7	>270	202	10.3	
Pneumococcal cap polysaccharide IgG2 (mg/L) (prevaccination)	ND	ND	35	8.1	82	ND	ND	
Pneumococcal cap polysaccharide IgG2 (mg/L) (postvaccination)	ND	51	>90	4.87	>90	ND	ND	
PHA (proliferation index)	4,560–86,042 (3–64%)	14,696 (11%)	4,114 (2%)	33,735 (29%)	1,700 (2%)	62,748 (34%)	ND	94,935–171,149 CPM
Con A (proliferation index)	20,476–117,727 (17–151%)	30,642 (37%)	31,984 (26%)	51,923 (60%)	6,665 (8%)	22,916 (22%)	ND	78,011–133,442 CPM
Tetanus toxoid (proliferation index)	1,018 (1%)	ND	337 (1%)	272 (1%)	ND	ND	ND	–
*Candida* (proliferation index)	883 (10%)	ND	1,397 (16%)	902 (3%)	ND	ND	ND	–
CARMIL2 mutation	L846Sfs*36	L846Sfs*36	L846Sfs*36	L846Sfs*36	L846Sfs*36	R50T	R50T	
Diagnosis method	WES	Targeted PCR	Targeted PCR	Genotyping and PCR	Genotyping and PCR	Genotyping and PCR	Genotyping and PCR	

*^a^Normal references for white blood cells counts (10^9^/L): for children 4–8 years, WBC: 5–14.5, neutrophils: 1.6–7.8, lymphocytes: 1.4–6.9, eosinophils: 0–0.43, and monocytes: 0.43–0.86, for children 9–12 years, WBC: 4.5–13.5, neutrophils: 1.4–8.2, lymphocytes: 1.2–6.4, eosinophils: 0–0.4, and monocytes: 0.40–0.8, for children 13–18 years, WBC: 4.5–13, neutrophils: 1.5–8.3, lymphocytes: 1.1–5.8, eosinophils: 0–0.39, and monocytes: 0.39–0.7, and for adults, WBC: 4.5–11, neutrophils: 1.6–7.2, lymphocytes: 1–4.8, eosinophils: 0–0.33, and monocytes: 0.33–0.6*.

*^b^Normal references for hemoglobin (g/L): 2–6 years: 115–155, 6–12 years: 115–155, 12–18 years: 130–160, and adults: 135–175*.

*^c^Normal references for lymphocytes markers for children 6–13 years of age: CD3: 1,700–1,900/mm^3^, CD3^+^/CD4^+^: 800–1,700/mm^3^, CD3^+^/CD8^+^: 700–1,000/mm^3^, and CD19^+^: 400–800/mm^3^, and CD16^+^/CD56^+^: 200–400/mm^3^. For 18–44 years of age: CD3: 782–2,834/mm^3^, CD3^+^/CD4^+^: 322–1,750/mm^3^, CD3^+^/CD8^+^: 338–1,086/mm^3^, CD19^+^: 67–555/mm^3^, and CD16^+^/CD56^+^: 200–400/mm^3^*.

*^d^Normal references for immunoglobulins: IgG (g/L): 1–6 years: 3.5–12.5, 6–12 years: 6.1–15.7, and 12 years adult: 7–16. For IgA (g/L): 3–6 years: 0.2–1, 6–12 years: 0.5–2, and 12 years adult: 0.4–2.3. For IgM (g/L): 3–9 years: 0.43–2.1, 9–12 years: 0.52–2.4, and 12 years adult: 0.4–2.3. For IgE (KU/L): 4–6 years: 8–250, 7–18 years: 8–350, adult: 5–500*.

*Proliferation index calculated as compared with two healthy control samples*.

*WBC, white blood cell count; EBV: Epstein–Barr virus; ND, not done; PHA, phytohemagglutinin T cell activation*.

F2P1 and F2P2 are two female siblings born to consanguineous Saudi parents. F2P1 is a 34 years old with history of recurrent skin abscesses and chest infections with secondary bronchiectasis since the age of 2 years. She had dysphagia secondary to *Candida* esophagitis. F2P2 is 28 years old with history of eczematous dermatitis and skin abscesses since age of 5 years. She had recurrent chest infections and bronchiectasis requiring pneumonectomy. EBV viremia was documented without any EBV-related clinical manifestation. Their immunological testing showed poor antibody response to tetanus and pneumococcal vaccines and poor T-cell response to mitogens and antigen stimulation (Table 1).

F3P1 and F3P2 are two male siblings born to consanguineous Saudi parents. F3P1 is 12 years old with eczematous dermatitis and recurrent skin abscesses since age of 3 months. He had recurrent chest infections, bronchiectasis, persistent supportive otitis media, scalp *Aspergillus niger* infection, nasal vestibulitis, and history of cervical lymphadenitis. In addition, he had disseminated skin warts (Figure 1A). He had high IgE level, and poor antibody response to tetanus vaccine (Table 1). He was suspected to have hyper IgE syndrome but *STAT3* and *DOCK8* gene sequencing were negative. F3P2 is a 4-year-old boy with early neonatal onset of eczematous dermatitis, skin abscesses, and chronic suppurative otitis media and chest infections. He had chronic diarrhea and dysphagia. Gastrointestinal endoscopy showed evidence of eosinophilic esophagitis and severe colitis with crypts abscesses. He also had disseminated skin warts and poor antibody response to tetanus vaccine (Table 1).

### Identification of Novel CARMIL2 Mutations

All families were recruited as part of our continuing efforts to identify the genetic causes of primary immunodeficiencies in consanguineous families from the Gulf region. All three families were unsolved but a higher priority was assigned to family F1 due to the presence of three affected individuals in the extended pedigree (Figure 1B). Initially, the index case of F1 was assessed using targeted next-generation sequencing, a gene panel that is specific for coding and splice site regions of 162 known PID genes (10). As the result was negative, and given the consanguineous structure of the pedigree, we proceeded with both WES of the affected cousin (F1P3) as well as genomewide high-density genotyping as a means of cataloging all autozygous intervals. The resulting WES data were filtered to focus on variants that resided within regions of autozygosity that were shared exclusively between the three affected members, and which were absent from our ethnically matched variant database (Figure 1D). After subjecting the exome capture data to all filters only one variant remained, a homozygous 13bp frameshift deletion in *CARMIL2* predicted to cause premature truncation of the protein shortly downstream of its midpoint (NM_001013838.1:c.2536_2548del:p.L846Sfs*36) (Figure 1E). The variant fully segregated with the disease state (Figure 1B).

Subsequent to this finding we interrogated our cohort of unsolved CID cases for additional families linking to this gene. Due to the high rate of consanguinity observed in our collection, we utilized high-density genotyping to search for other cases with exclusive autozygous intervals that encompassed the *CARMIL2* locus. Our search uncovered two additional families (Figures 1B,C). In family F2, two adult sisters who were being followed for B cell defects and hypogammaglobulinemia were found to carry the same homozygous mutation as confirmed through Sanger sequencing. Although unrelated, the two families shared the same haplotype (Figure S1 in Supplementary Material), raising the possibility of a founder effect. In family F3, two boys with a suspected hyper-IgE-like condition mapped to the locus but with a dissimilar haplotype. Sanger sequencing of all coding and splice site regions in *CARMIL2* revealed a missense variant near the protein’s N-terminus (NM_001013838.1:c.149G>C:p.R50T) (Figure 1E). This mutation segregated with the disease state and was not present in the ExAC Browser or the Greater Middle East Variome (14). It was likewise absent from our local databases. Multiple *in silico* algorithms strongly predicted pathogenicity for this amino acid change (Table S1 in Supplementary Material), and the affected residue was confirmed to be very highly conserved across species down to the level of Xenopus and *C. elegans* (Figure 2A). Among the three proteins that make up the CARMIL family of genes, this residue is fully conserved (Figure S2 in Supplementary Material).

### CARMIL2 Mutations Cause RNA and Protein Level Changes

Due to the truncating nature of the mutation in families F1 and F2, we anticipated that nonsense-mediated RNA decay (NMD) surveillance may act upon the aberrant transcripts. To check this we performed real-time RT-PCR on patient lymphoblastoid RNA and observed a highly significant drop of >75% in CARMIL2 expression levels for the F1 proband versus healthy controls (*p* < 0.0001). This strongly suggested that NMD was acting to remove these mutant transcripts due to the presence of a premature stop codon. Somewhat surprisingly we also detected a significant (*p* < 0.02) expression decrease of approximately 35% between the F3 index case (p.R50T) and our controls (Figure 2C).

Finally, to assess how the two mutations were affecting protein expression and stability we performed an immunoblot and probed with an anti-RLTPR/CARMIL2 antibody that recognized an epitope upstream of the truncation site. As expected for the frameshift mutation, our data did not reveal any full-sized CARMIL2 protein in the F1 lymphoblastoid cells. We calculated the estimated molecular weight for the truncated form of the protein but failed to see any corresponding band of that size on our Western (Figure 2B, Figure S3 in Supplementary Material). Similarly there was a loss of detectable CARMIL2 for the F3 patients, indicating that the p.R50T missense mutation was leading to gross instability of the protein. It is worth noting, however, that the CARMIL2 band intensity varied considerably between our normal controls, and therefore residual protein expression (as well as weak expression of a truncated form) in our patients cannot be ruled out.

### 3D Modeling Suggests Disturbed Structural Stability

Two 3D homology models were constructed using SWISS-MODEL, one containing the native Arg-50 residue and the other the mutant Thr-50 (Figure 2D). Exchanging a positively charged, polar amino acid for another which is considerably smaller and uncharged may introduce voids which potentially destabilize the surrounding three-dimensional structure, as well as generating perturbations in the electrostatic potential distribution. Hence, an energy computation for the native and mutant residues was done with the GROMOS96 43B1 parameters set, without reaction field. The native Arg-50 exhibited a total *E* = −279.218 while the mutant Thr-50 showed *E* = 5.683, a drastic increase in electrostatic energy. By expanding our focus to include all residues within 5 Å of Arg-50, we found additional amino acids whose energy levels were predicted to be perturbed (Table S2 in Supplementary Material). These calculations suggest electrostatic instability within the peptide, as supported by the lack of detectable mutant protein following the immunoblot.

### Immunophenotyping of CARMIL2-Deficient T Cells

**Figure 3:**
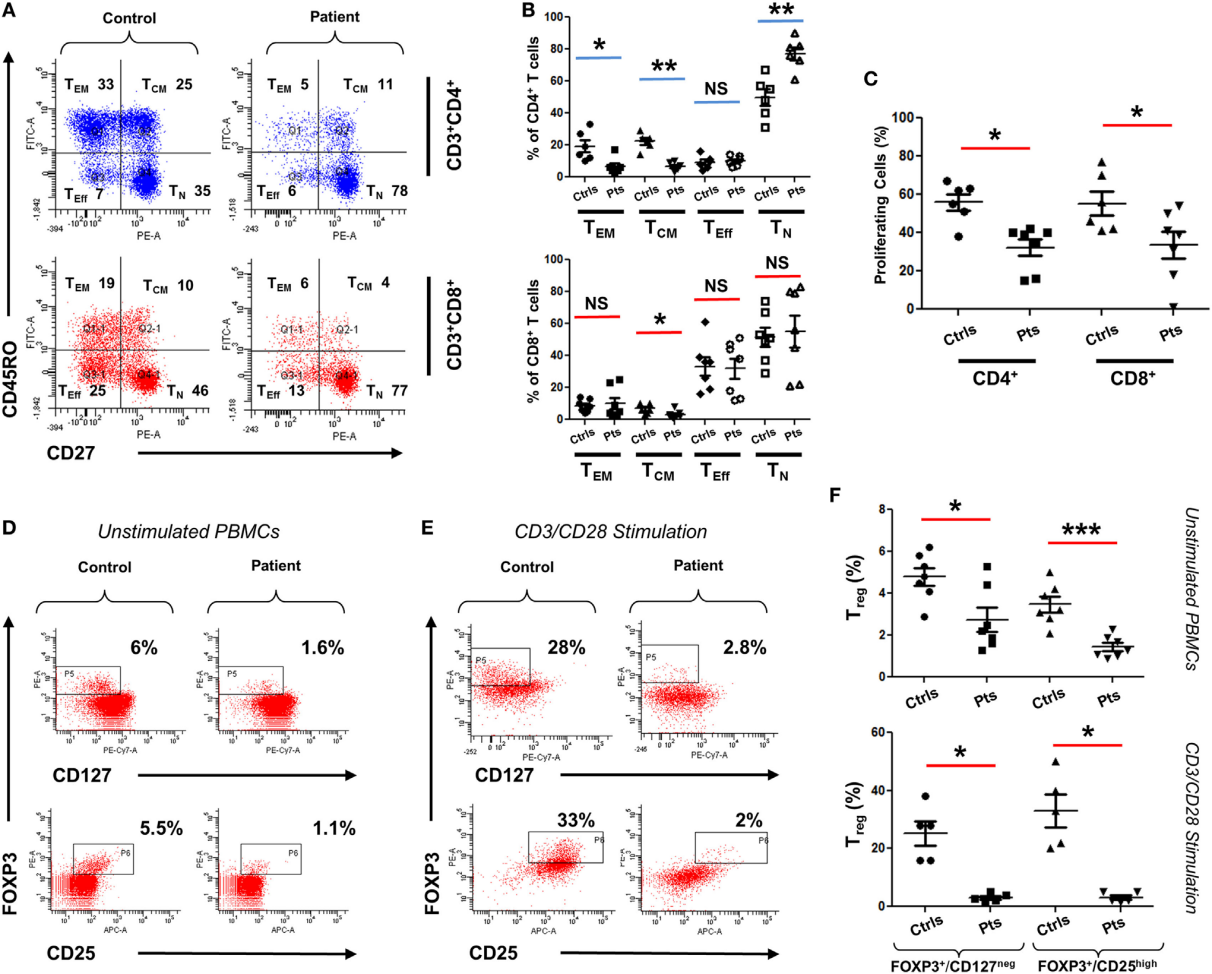
Immunophenotyping of CARMIL2-deficient T cells. **(A)** Representative plot of viable PBMCs from one control and one patient, gated as shown then stained with CD45RO/CD27 to determine T cell subtype population numbers. Plots show CD4 and CD8 T cells that are naïve (T_N_, CD45RO^−^CD27^+^), central memory (T_CM_, CD45RO^+^CD27^+^), effector memory (T_EM_, CD45RO^+^CD27^−^), and effector (T_Eff_, CD45RO^−^CD27^−^). Corresponding percentages are indicated in each quadrant. **(B)** Summary of CD45RO/CD27 staining data from CARMIL2 patients (*n* = 7), along with healthy controls (*n* = 6), separated based on CD4/CD8 subtype. Asterisks indicate significance levels (*p < 0.05, **p < 0.01, and ***p < 0.001) and NS denotes not significant. **(C)** Summary of CFSE proliferation data on PBMCs from controls and patients, following CD3/CD28 stimulation for 72 h. Representative plots showing T_reg_ cell counts on a control and a patient, using unstimulated **(D)** and 72 h-stimulated **(E)** PBMCs. Viable cells were gated on CD4^+^ and were stained with FOXP3/CD25 and FOXP3/CD127 as indicated. Corresponding percentages are provided in each square. **(F)** Summary of T_reg_ cell count data for unstimulated as well as CD3/CD28-stimulated groups. Data are separated based on FOXP3^+^/CD127^low^ or FOXP3^+^/CD25^high^ staining.

To functionally assess the immunological consequences of CARMIL2 deficiency in our patients, peripheral blood mononuclear cells (PBMCs) from patients and controls were subjected to CD45RO/CD27 staining. This allowed us to ascertain the percentage of each T cell subtype in the absence of any external stimuli. Although CD8^+^ T cells did not exhibit significant deviation between controls and patients (except in the case of central memory cells), CD4^+^ cells did exhibit significant changes in percentages including a marked skewing toward the naïve form (Figures 3A,B). Next we directly tested cellular response to CD3/CD28 stimulation using a CFSE proliferation assay, and found that after 72 h of stimulation both CD4^+^ and CD8^+^ patient cells were significantly lagging behind their normal control counterparts (Figure 3C), indicating that our CARMIL2 mutations were causing an impaired response to CD3/CD28 similar to that observed in other patients (5, 7). Among the most constant immunological findings in previous studies has been a decrease in overall patient regulatory T cell (T_reg_) numbers, so we also sought to characterize this immunological phenotype using dual FOXP3/CD25 and FOXP3/CD127 staining (Figures 3D,E). T_reg_ cells were consistently depressed in our patients, whether measured directly or after activation of PBMC with CD3/CD28 stimulation (Figure 3F).

## Discussion

Here, we report seven patients from three apparently unrelated consanguineous Saudi families who presented with varying symptoms of CID and were found to harbor mutations in CARMIL2. Eczematous, dry, and scaly dermatitis associated with recurrent skin abscesses was the commonest clinical presentation among all affected patients. *S. aureus* was frequently isolated from skin abscesses. Interestingly, skin disease is variable between patients, ranging from severe eczematous-like rash to mild dry skin disease. Recurrent upper and lower respiratory infections were observed in five of the seven patients with secondary bronchiectasis and chronic lung diseases. Most of our patients had poor specific antibody response to protein antigens like tetanus vaccine and a few to polysaccharide antigens. Although most of our patients had severe T-cell dysfunction as evident from their poor response to mitogen and antigen stimulations, they did not suffer from unusual severe viral or opportunistic infections. Asymptomatic EBV viremia was observed in two patients, and this suggests that they may be prone to EBV-driven clinical manifestations in the future as has been previously reported (7). A striking feature of our CARMIL2-deficient patients is their esophageal disease as was observed in five patients, mostly secondary to *Candida* infection, eosinophilic or nonspecific active esophagitis. In addition one patient had chronic diarrhea secondary to severe colitis. Although our patients have deficient T_reg_ counts, the only evidence of immune dysregulation was increased IgE level in two of our patients (F103 and F3P1), food allergy, asthma and allergic rhinitis in patients F3P1 and F3P2.

Recently, three independent reports have linked autosomal recessive mutations in this gene to a new form of CID (5–7). The patients hailed from multiple countries (Morocco, Tunisia, Turkey, Norway, Yemen, and Brazil) and demonstrated the presence of modifier effects with relation to CARMIL2 mutations, since patients with the same mutation were shown to exhibit differences in clinical presentation. Although a key immunological finding in those patients (as well as ours) was loss of circulating T_reg_ cells, autoimmune manifestations were surprisingly absent. In this regard CARMIL2 patients are similar to those with BCL10 and CARD11 deficiencies. In all three cases depressed T_reg_ numbers but lack of autoimmunity are coupled with a CD28 co-stimulation defect (15, 16).

With the seven patients we are presenting here, the cohort of all CARMIL2 cases reported worldwide increases to twenty one cases. Table 2 summarizes the common clinical features of published CARMIL2-deficient patients. It is interesting to note that consanguinity as a predisposing factor is reported in 76% of cases. The most common clinical presentations across all studies are dermatitis (80%), recurrent chest infections (80%), and skin abscesses (62%). Persistent uncontrolled viral infections like warts, molluscum contagiosum, and EBV are also common. CARMIL2 defect is also a predisposing factor to severe mucocutaneous candidiasis and mycobactrial infections.

**Table 2:** Common clinical features of all reported CARMIL2-deficient patients.

	Sorte et al. (6)	Wang et al. (5)	Schober et al. (7)	This study	Total
Total patients	4	6	4	7	21
Age range (years)	18–52	2–27	4–14	4–34	2–52
Sex	2M/2F	2M/4F	2M/2F	4M/3F	10M/11F
Consanguinity	0/4	5/6	4/4	7/7	16/21 (76%)
Dermatitis	4/4	5/6	2/4	6/7	17/21 (80%)
Recurrent chest infections	3/4	5/6	4/4	5/7	17/21 (80%)
Skin abscesses	3/4	2/6	1/4	7/7	13/21 (62%)
Warts	4/4	0/6	2/4	2/7	8/21 (38%)
Poor antibody response		2/6		6/7	8/13 (61%)
Esophageal disease	0/4	1/6	0/4	5/7	6/21 (29%)
Mucocutaneous candidiasis	0/4	3/6	0/4	2/7	5/21 (24%)
Epstein–Barr virus-related smooth muscle tumor	0/4	0/6	4/4	0/7	4/21 (19%)
Molluscum contagiosum	2/4	1/6	0/4	0/7	3/21 (14%)
Mycobacterial infections	0/4	2/6	0/4	0/7	2/21 (9%)
Mortality	0/4	1/6	3/4	0/7	4/21 (19%)

CARMIL2, also known as RLTPR, is a regulator of actin capping protein which is a critical component of cell motility. It is part of the human CARMIL family, of which at least the first two members have distinct cell migration functions that are nonredundant (17). In fibrosarcoma cells CARMIL2 provides the molecular link between vimentin intermediate filaments and actin, thereby playing a role in cell polarity, lamellipodial assembly, and the creation of protrusions that allow cellular migration (18). T cell lymphoblasts which are CARMIL2-deficient exhibit poor actin distribution along the leading edges along with severely impaired microtubule networks. In migration experiments these cells move with increased speed but are less capable of chemokine-directed movement, demonstrating that this protein is critical for T cell cytoskeletal organization (7).

CARMIL2 deficiency also impairs the canonical NF-κB pathway in patient T lymphoblasts, although phosphorylation of upstream (ZAP70) and downstream (ERK1/2) proteins remains intact (7). Intriguingly CARMIL2 can also work in a dominant mode, as a gain-of-function mutation (Q575E) has been linked to cutaneous T cell lymphoma, selectively upregulating the NF-κB pathway and causing activated T cells to significantly increase their IL-2 production (19).

Among the domains present in CARMIL2 is a leucine-rich repeat (LRR). LRR domains contain a common motif of 20–30 amino acids that is highly conserved between species, and they play a role in protein–protein interactions. In the case of CARMIL2, the LRR allows targeting of the peptide to vimentin intermediate filaments (18). The repetitive motif sequence forms a curved solenoid structure, which derives stability from a hydrophobic inner core that is rich in leucines (20). All the CARMIL2 autosomal recessive missense mutations reported previously have involved leucine residues that are present within the LRR domain (Figure 2E), suggesting that alteration of these critical leucines disrupts the stabilizing hydrophobic core of the horseshoe LRR structure (Figure 2F). Our patients from family F3 reveal a missense alteration of an arginine located at the N-terminal pleckstrin homology (PH) domain. PH domains are evolutionarily conserved and often mediate localization to the membrane, which is the case with CARMIL1, although in CARMIL2 membrane targeting is achieved through a separate C-terminal stretch of 27 amino acids and the PH domain is thought to be involved with LRR in anchoring the protein to filaments (21). Our energy computations for 3D homology models (native and mutant) revealed a drastic increase in electrostatic energy for the mutant Thr-50 residue along with changes to several surrounding amino acids, increasing the overall calculated energy for the peptide and rendering it unstable.

In summary, the seven patients we report here represent the first documented cases from the Gulf region and exhibit a similar degree of phenotypic variability between individuals with the same mutation as was observed in other populations. This is underscored by the fact that these families were initially classified as CID, B cell class switch defect, and DOCK8-suspected hyper IgE, respectively. This is a reflection of both the wide-ranging molecular roles played by CARMIL2, as well as the presence of modifying factors affecting each individual’s clinical presentation.

## Ethics Statement

All research involving patient materials was approved by the institutional review board at KFSHRC, RAC # 2080 025. Written consent was obtained from patients or their guardians and supported by the NSTIP strategic technologies program of Saudi Arabia (KACST 14-MED316-20).

## Author Contributions

AA and HA-M designed the study, analyzed data, and prepared the manuscript; SA and HA performed experiments and assisted in data analysis; HG performed the flow cytometry experiments including data analysis and participated in manuscript writing; MA-H, BA-S, RA, HA-D, FS, and HA-M cared for patients and contributed the clinical data; NA-N carried out the 3D modeling, *in silico* analysis and participated in manuscript writing; DM, ZS, and MA generated the WES data and provided bioinformatic support.

## Conflict of Interest Statement

The authors declare that the research was conducted in the absence of any commercial or financial relationships that could be construed as a potential conflict of interest.

## Footnotes

^1^www.ebi.ac.uk/arrayexpress.

^2^http://genome.ucsc.edu/.

^3^www.ebi.ac.uk/ena.
