# Supplementary material for: Novel CARMIL2 Mutations in Patients with Variable Clinical Dermatitis, Infections, and Combined Immunodeficiency
Source: Front Immunol. 2018 Feb 9;9:203. doi: 10.3389/fimmu.2018.00203 (PMC5811477; doi:10.3389/fimmu.2018.00203)
Supplement: Supplementary file 1 [file data_sheet_1.docx]

SUPPLEMENTARY FIGURE S1

**F1P1 F1P2 F1P3 F2P1 F2P2**


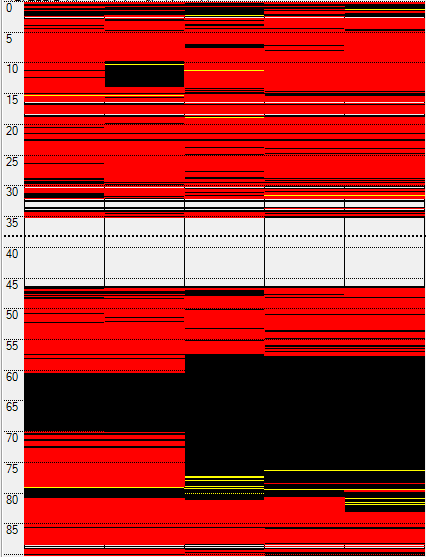

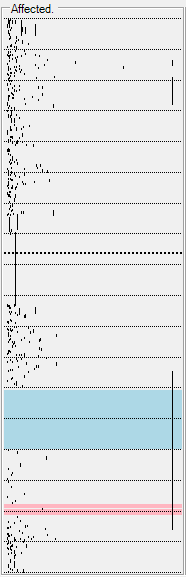


*CARMIL2*

SUPPLEMENTARY FIGURE S2


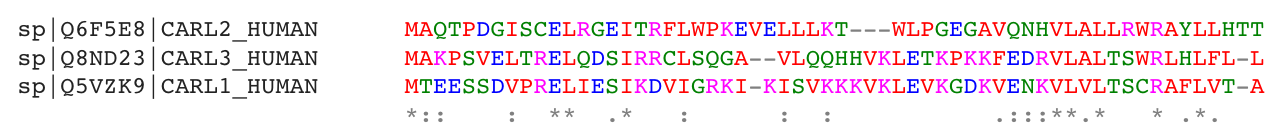


SUPPLEMENTARY FIGURE S3

**C1 C2 C3 F3P1 F3P2 F1P1 F1P2**
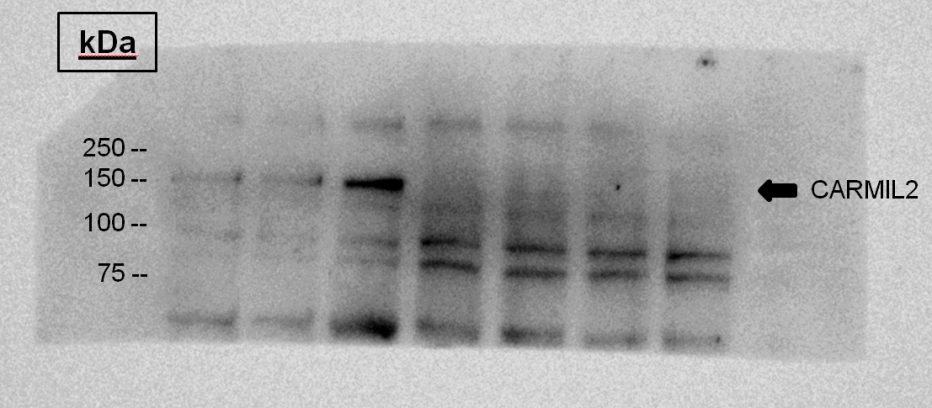


Figure Legends.

Supp. Fig. S1. Haplotypes for affected individuals from families F1 & F2, generated using the AxiomGWH SNP Chip and the AutoSNPa software. Heterozygous SNPs are red and regions of homozygosity are indicated as black. The window on the right highlights areas that have shared homozygosity across all F1 & F2 affecteds with the same (pale blue) as well as different (pink) haplotypes.

Supp. Fig. S2. Sequence alignment for the three peptides which form the CARMIL family of proteins, indicating that the mutated Arg50 residue (arrowhead) in CARMIL2 is conserved across all members of this family.

Supp. Fig. S3. The immunoblot pictured in Fig. 2B, showing the entire membrane area that was probed with anti-CARMIL2. Size indicators are given to the left.

SUPPLEMENTARY TABLES

Table S1. *In silico* analysis of the CARMIL2 missense mutation

| **Protein Change** | **Algorithm** | **Score** | **Prediction** |
| --- | --- | --- | --- |
| CARMIL2 (p.R50T) | MutationTaster | 71 | Disease causing |
|  | Polyphen-2.0 | 0.991 | Probably damaging |
|  | SIFT | 0.000 | Damaging |
|  | PROVEAN | -4.77 | Deleterious |
|  | CONDEL | 0.527 | Damaging |

Table S2. Computed electrostatic energies for all residues within 5.0 Å of R50

| **Residue** | **Native protein** | **Mutant protein** |
| --- | --- | --- |
| Ala-45 | -39.436 | -39.536 |
| Leu-46 | -59.729 | -59.731 |
| Leu-47 | -32.001 | -31.531 |
| Arg-48 | -243.374 | -243.086 |
| Trp-49 | -32.931 | -16.165 |
| Arg-50 | -279.218 | +5.683 |
| Ala-51 | -38.082 | -31.147 |
| Tyr-52 | -77.544 | -77.646 |
| Thr-66 | -18.615 | -14.440 |
| Phe-67 | -40.936 | -38.318 |
| Ser-68 | -35.619 | -37.406 |
| Tyr-69 | -82.829 | -84.549 |
| Glu-71 | -32.702 | -32.241 |
| Asp-167 | -4.469 | +7.993 |
| Tyr-168 | -50.345 | -48.730 |
